# CARE NETWORKS OF RURAL APPALACHIAN FAMILY CAREGIVERS AND PERSONS LIVING WITH DEMENTIA

**DOI:** 10.1093/geroni/igac059.776

**Published:** 2022-12-20

**Authors:** Jyoti Savla, Karen Roberto, Rosemary Blieszner

**Affiliations:** Virginia Tech, Blacksburg, Virginia, United States; Virginia Polytechnic Institute & State University, Blacksburg, Virginia, United States; Virginia Tech, Blacksburg, Virginia, United States

## Abstract

Dementia care research typically focuses on primary family caregivers with limited consideration of their engagement with a broader care network and the geographic area in which they reside. Using a mixed-methods approach, we analyzed care networks of 163 primary caregivers and their relative living with dementia in rural Virginia. Six distinct care network types emerged based on the primary caregiver's gender, relationship to the person living with dementia, and presence of other informal caregivers. Networks differed by the caregiver’s emotional connectivity with family/friends and feelings of caregiver strain, role overload, and loneliness. Caregivers’ service use attitudes and support service utilization varied across network types and across divergent economic resources of the rural counties in which families resided. Findings establish a framework for understanding the types and influences of care networks and tailoring services to support dementia family caregivers in diverse rural areas.

